# Multi-Omics Analysis Revealed the Accumulation of Flavonoids and Shift of Fungal Community Structure Caused by Tea Grafting (*Camellia sinensis* L.)

**DOI:** 10.3390/plants14081176

**Published:** 2025-04-10

**Authors:** Yue Liu, Jun Liu, Yiping Tian, Shuang Ye, Dandan Pang, Linbo Chen, Hao Qu

**Affiliations:** 1 Tea Research Institute, Yunnan Academy of Agricultural Sciences, Kunming 650000, China; liuyue0504@126.com (Y.L.); ckstyp@163.com (Y.T.); yeshuang910@163.com (S.Y.); pangddnumber1@163.com (D.P.); 2 Yunnan Provincial Key Laboratory of Tea Science, Tea Research Institute, Yunnan Academy of Agricultural Sciences, Menghai 666201, China; 3 Bioinformatics Group, Wageningen University & Research, 6708 PB Wageningen, The Netherlands; ahjunnew@163.com

**Keywords:** tea, grafting, metabolic profile, flavonoids, secondary metabolites

## Abstract

Grafting is an important approach to improving tea plant varieties, and tea grafting can result in changes in secondary metabolites. However, the shifting pattern of secondary metabolites between scions, rootstocks, and non-grafted tea leaves is unclear. We employed “Yuncha 1” as the scion and “Zijuan” as the rootstock with significant differences in leaf color traits to reveal the influences of grafting on the generation of secondary metabolites and transcriptional activities. By non-targeted metabolomic analysis, we identified that grafting led to an obvious shift in secondary metabolites between the scion, rootstock, and non-grafted tea leaves. Importantly, we found that grafting significantly enhanced the accumulation of flavonoids, the vital component of secondary metabolites for the flavor quality, purple color, and health-beneficial effect of Zijuan tea. Via transcriptomics, we found that the key biosynthetic genes *DFR* and *EC 1.1.1.219* for synthesizing flavonoids were significantly enhanced in rootstock compared with non-grafted Zijuan. Concurrently, *ANS* for biodegrading flavonoids was significantly suppressed in rootstock compared with non-grafted Zijuan. These results revealed the shifting mechanism of key secondary metabolites during grafting. In addition, we found that the shift after grafting possessed no significant influence on bacterial community diversity, but grafting slightly enhanced the fungal community diversity of scions. We found that the shift in fungal community diversity was driven by rootstocks with a higher fungal community diversity. This study systematically reveals the shift in secondary metabolites and fungal community diversity, which provides a novel and comprehensive understanding and theoretical basis for plant breeding using grafting.

## 1. Introduction

Tea is one of the oldest and most popular beverages all over the world [1,2]. The production of tea involves the growing of tea plants and the processing of tea leaves [3]. The quality, characteristics, and yield of tea are influenced by various factors, including genetic traits of the tea tree, cultivation conditions, and the processing of tea leaves [3,4]. Desirable tea planting needs tea trees to possess a typical metabolic profile, high yield, and high disease resistance [5]. However, each tea species has its advantages and disadvantages due to the genetic element [6,7]. Therefore, it is vital to achieve different advantages and overcome the disadvantages of tea planting.

Grafting has been employed to enhance the performance of tea trees regarding flavor and secondary metabolites [8,9]. Grafting can increase the content of volatile metabolites including geraniol, phenylethyl alcohol, (E)-nerolidol, decanal, and linalool oxides of the final product of tea [8]. In fresh tea leaves, grafting can influence the formation of secondary metabolites such as phenolic acids, flavan-3-ols, dimeric catechins, flavanol, and flavanol/flavone glycosides [10]. Cheng et al. found that grafting induces an increase in secondary metabolites including flavonoids and catechins in the leaves of scion and rootstock by the upregulation of biosynthesis pathways [11]. However, the shifting pattern and transcriptional mechanism of the secondary metabolism of fresh tea leaves driven by drafting are not well characterized.

In addition, tea leaf secondary metabolites play an important role in microbial community diversity. The composition of microbial communities on tea leaves varies with tea varieties and their polyphenol content. Yao et al. found that tea varieties with higher polyphenol levels (>20%) host distinct endophytic and rhizosphere microbial communities, with leaf endophytes showing greater separation in high-polyphenol cultivars [12]. This suggests that secondary metabolites significantly influence the assembly of the microbial community of tea trees. Although the effects of tea grafting on the generation of secondary metabolites and the influence of secondary metabolites on tea microbial communities have been explored, the correlation between tea grafting, the formation of secondary metabolites, and the assembly of tea phyllosphere microbial communities remains unknown. Filling this knowledge gap will provide a fundamental understanding for practicing tea breeding by grafting.

**Figure 1:**
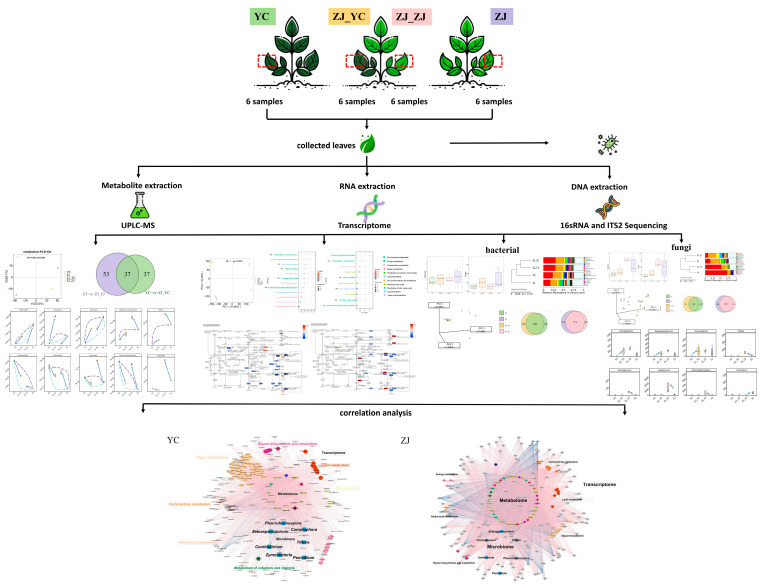
Technical workflow of the study. It shows the collection of fresh tea leaves from four different groups, followed by metabolite extraction analyzed by UPLC-MS, RNA extraction analyzed by transcriptome sequencing, and DNA extraction of leaf microbiome analyzed by 16S rDNA and ITS2 amplicon sequencing. Correlation analysis was performed on the combined data to understand the effects of grafting on tea leaf characteristics.

In this study, we used Zijuan (ZJ) and Yuncha1 (YC) to explore the effects of tea grafting on the generation of secondary metabolites, the transcriptional profile, and the tea phyllosphere microbiome. ZJ, one of the most well-known representatives of tea plants with extraordinary purple shoots and normalized fourth and fifth leaves [13,14], was used as the rootstock. YC, a tea with strong fertility, good tenderness retention, dense germination, and abundant hairy hairs [14,15], was used as the scion for the grafting operation (Figure 1 and Figure S1). This study employed multi-omics, integrating fresh tea leaves and microbiota to gain a comprehensive understanding of the influence of grafting on the tea leaf metabolome, transcriptome, and microbiome. Firstly, we employed the non-targeted metabolomic analysis based on ultra-high performance liquid chromatography–mass spectrometry (UHPLC–MS) to characterize the effects of grafting on the non-volatile metabolites of tea leaves. Then, the transcriptomics of tea leaves were employed to reveal how grafting influences the transcriptional profile of secondary metabolites. After that, the effects of grafting on the tea phyllosphere microbial community were uncovered by 16S rDNA and ITS2 amplicon sequencing. Finally, the effects of the transcriptome and microbial communities on the generation of secondary metabolites after tea grafting were systematically studied through multi-omics integration. This study will provide theoretical knowledge for practicing grafting in tea cultivation and breeding.

## 2. Results

### 2.1. Metabolic Profile Shift in Tea Leaves After Grafting

Metabolomics analysis revealed distinct shifts in the metabolic profiles of tea leaves following grafting, identifying 465 known metabolites across various functional categories (Table S1). Among these, flavonoids dominated as the most abundant group, comprising 93 metabolites (20.0%), followed by polyketides with 56 metabolites (12.0%). Other notable categories included terpenoids (43 metabolites, 9.2%), benzene and derivatives (32 metabolites, 6.9%), fatty acyls (28 metabolites, 6.0%), and carbohydrates (25 metabolites, 5.4%). These metabolites were mapped to distinct metabolic pathways (Figure S2), with the “biosynthesis of other secondary metabolites” pathway being the most enriched (77 metabolites), followed by “carbohydrate metabolism” (37 metabolites) and the “metabolism of terpenoids and polyketides” (34 metabolites).

**Figure 2:**
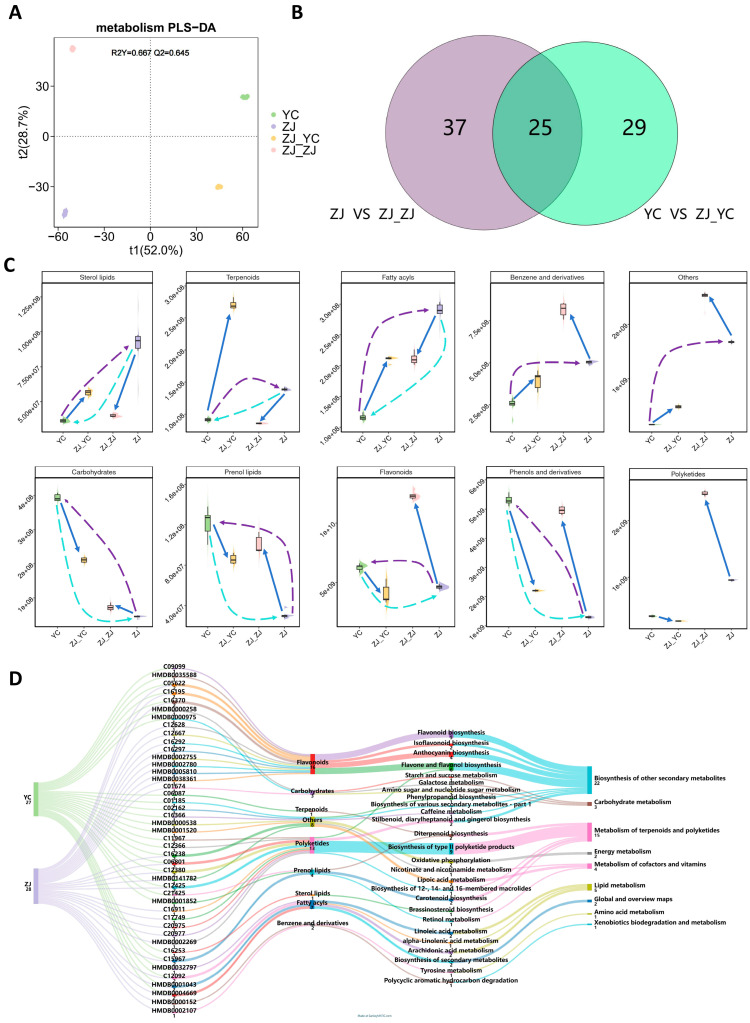
Metabolomic analysis of fresh tea leaves before and after grafting. (**A**) PLS-DA plot of metabolomics data showing the separation of metabolites among the YC, ZJ, ZJ_YC, and ZJ_ZJ groups. (**B**) Venn diagram comparing the number of differential metabolites before and after grafting in Yuncha (YC vs. ZJ_YC) and Zijuan (ZJ vs. ZJ_ZJ). (**C**) Boxplots comparing the levels of differential metabolites in various categories (flavonoids, polyketides, terpenoids, benzene and derivatives, fatty acyls, phenols and derivatives, prenol lipids, sterol lipids, carbohydrates, and others) among different groups. Solid arrows indicate the trend of changes from pre-grafting to post-grafting. The curve represents the trend of metabolite changes. The purple dashed line indicates the driving force for increased metabolites, while the blue dashed line indicates the driving force for decreased metabolites. (**D**) Sankey diagram showing the pathways and classification of differential metabolites before and after grafting of Yuncha (YC vs. ZJ_YC) and Zijuan (ZJ vs. ZJ_ZJ).

Further analysis highlighted significant metabolic divergence among the four sample groups (Figure 2A), with 91 differential metabolites identified in grafted tea plants (Table S2). Comparing specific groups, ZJ vs. ZJ_ZJ showed 41 significantly altered known metabolites, outnumbering the 37 identified in YC vs. ZJ_YC, with 25 metabolites shared between these comparisons (Figure 2B). To elucidate grafting-induced changes, we examined the variation patterns of these differential metabolites by compound type. In scions (ZJ_YC), grafting markedly increased the levels of sterol lipids, terpenoids, fatty acyls, and benzene derivatives. Conversely, rootstock leaves (ZJ_ZJ) exhibited elevated levels of secondary metabolites, including carbohydrates, phenol lipids, flavonoids, phenols, and polyketides (Figure 2C). Intriguingly, grafting appeared to promote a metabolic balancing effect: metabolites initially higher in scions (e.g., sterol lipids, terpenoids, prenol lipids, and flavonoids) decreased post-grafting, while increasing in rootstocks, suggesting a coordinated metabolic adjustment between scion and rootstock.

Focusing on secondary metabolites, 55 compounds displayed significant differences across key metabolic pathways (Figure 2D). These included the “metabolism of terpenoids and polyketides” (fifteen compounds), “lipid metabolism” (five compounds), “carbohydrate metabolism” (three compounds), “metabolism of cofactors and vitamins” (four compounds), and “energy metabolism” (two compounds). Notably, flavonoids, with 16 differentially abundant compounds, emerged as the most significantly altered secondary metabolites. These flavonoids were distributed across pathways such as flavonoid biosynthesis, isoflavonoid biosynthesis, anthocyanin biosynthesis, and flavone/flavanol biosynthesis (Figure 2D), underscoring their potential role in shaping the flavor and quality of grafted tea. These metabolic shifts provide a foundation for exploring the molecular mechanisms driving these changes, as detailed in the subsequent transcriptional analysis.

### 2.2. Transcriptional Activity Driven by Grafting

**Figure 3:**
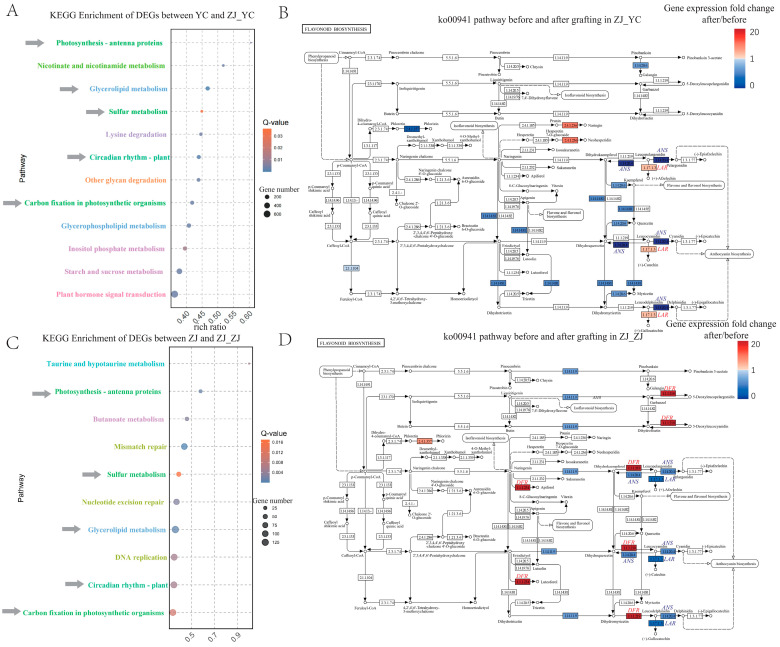
Transcriptomic comparison analyses. (**A**) KEGG pathway enrichment analysis of DEGs between YC and ZJ_YC. The size of the dots indicates the number of genes involved, while the color gradient reflects the Q-value of enrichment. Arrows indicate significantly enriched pathways in both YC and ZJ after grafting. Different pathway name colors represent the higher-level functional classifications of pathways. (**B**) Differential gene expression in the ko00941 pathway before and after grafting in ZJ_YC. (**C**) KEGG pathway enrichment analysis of DEGs between ZJ and ZJ_ZJ. The size of the dots indicates the number of genes involved, while the color gradient reflects the Q-value of enrichment. Arrows indicate significantly enriched pathways in both YC and ZJ after grafting. Different pathway name colors represent higher-level functional classifications of pathways. (**D**) Differential gene expression in the ko00941 pathway before and after grafting in ZJ_ZJ.

To uncover the molecular basis of the observed metabolic alterations, we investigated transcriptional changes in grafted tea plants, revealing pronounced differences in gene expression profiles (Figure S3A). In scions, compared to ungrafted controls, 8558 genes were upregulated and 8493 were downregulated (Figure S3B). Similarly, rootstock leaves exhibited significant transcriptional reprogramming, with 6918 genes upregulated and 6795 downregulated (Figure S3C). These differentially expressed genes (DEGs) were associated with metabolite-related pathways and displayed distinct expression patterns (Figure S3D). Shared pathways with differential changes in both scion and rootstock included photosynthesis antenna proteins, sulfur metabolism, glycerolipid metabolism, carbon fixation, and circadian rhythm (Figure 3A,C). Paralleling the metabolomic findings, gene expression showed a balancing trend post-grafting: genes initially more expressed in scions than rootstocks decreased in scions and increased in rootstocks, particularly those linked to key flavor compounds like flavonoids.

A deeper analysis of the flavonoid biosynthesis pathway (ko00941) revealed contrasting regulatory patterns. In scions (ZJ_YC), many genes were significantly downregulated (Figure 3B), while in rootstocks (ZJ_ZJ), numerous genes were upregulated (Figure 3D). For instance, the key gene *DFR* (K13082), encoding flavanone 4-reductase for synthesizing apiforol and luteoforol, was dramatically upregulated in ZJ_ZJ (*p* < 0.05, fold change > 20) compared to ZJ. Additionally, DFR encoding bifunctional dihydroflavonol 4-reductase was significantly enhanced, boosting the biosynthesis of leucodelphinidin, leucocyanidin, and leucopelargonidin. These transcriptional changes align with metabolomic data, where flavonoids decreased in ZJ_YC but increased in ZJ_ZJ post-grafting. Conversely, genes such as *ANS* (K05277) and *LAR* (K13081), encoding anthocyanidin synthase [EC:1.14.20.4] and leucoanthocyanidin reductase [EC:1.17.1.3], respectively, were significantly suppressed in ZJ_ZJ (*p* < 0.05) compared to ZJ (Table S3), potentially fine-tuning flavonoid diversity. These findings demonstrate that grafting triggers a complex interplay of metabolic and transcriptional reprogramming, with flavonoids serving as a critical link between these processes.

### 2.3. Shift in Microbial Communities’ Diversity by Grafting

To investigate how grafting influences microbial communities and their potential effects on scion secondary metabolites, we analyzed bacterial and fungal diversity in tea leaves. Grafting exerted a minimal impact on bacterial community diversity (Figure S4A,B), yet it significantly altered the abundance of specific bacterial genera. In grafted scions (ZJ_YC), genera such as *Blautia*, *Phascolarctobacterium*, and *Spirosoma* increased in abundance, while *Aureimonas*, *Bosea*, *Mucilaginibacter*, and *Pedobacter* decreased. Notably, *Methylobacterium* abundance rose in both scions and rootstocks post-grafting (Figure S4C). Grafting also reshaped bacterial genus composition: scions gained 58 new genera and lost 36 compared to ungrafted leaves, while rootstocks acquired 30 new genera and lost 54 (Figure S4D). Specific genera unique to grafted scions (ZJ_YC), such as *Atopobium*, *Megasphaera*, *Methylocella*, *Nitrosospira*, and *Rubellimicrobium*, were detected in rootstocks but absent in ungrafted YC. Similarly, genera like *Campylobacter*, *Curtobacterium*, *Fusicatenibacter*, *Massilia*, and *Pygmaiobacter* appeared in rootstocks (ZJ_ZJ) but were absent in ungrafted ZJ, suggesting microbial exchange or adaptation induced by grafting.

**Figure 4:**
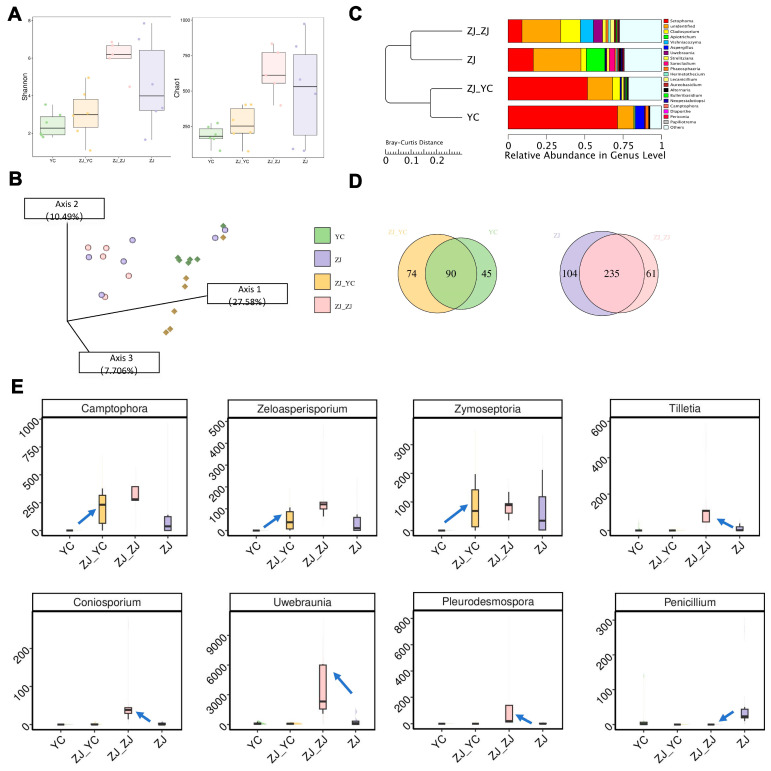
Microbiota diversity comparison analysis by ITS2. (**A**) Alpha diversity indices (Shannon and Chao1) boxplot. (**B**) Principal Coordinates Analysis (PCoA) plot based on Aitchison distance. The circle represents YC, and the square represents ZJ. (**C**) Hierarchical clustering and relative abundance barplots of top 20 at the genus level. (**D**) Venn diagrams displaying the number of genera between YC and ZJ_YC and between ZJ and ZJ_ZJ. (**E**) Boxplots of significantly different genera in their abundance between grafted and non-grafted groups based on ANCOM and LEfSe. The differential genera shown here are common to both methods. The arrows indicate a significant directional change.

In contrast, grafting significantly enhanced fungal community diversity in both scions and rootstocks (Figure 4A,B). Analysis of the top 20 fungal genera revealed marked shifts in abundance (Figure 4C). Scions gained 74 new fungal genera and lost 45 compared to ungrafted leaves, while rootstocks added 61 genera and lost 104. Despite these changes, a core fungal community persisted, with 90 genera shared between YC and ZJ_YC, and 235 between ZJ and ZJ_ZJ, outnumbering unique genera (Figure 4D). Using ANCOM and LEfSe analyses, we identified significant abundance differences: in scions (ZJ_YC), genera such as *Camptophora*, *Zeloasperisporium*, and *Zymoseptoria* were enriched compared to YC (Figure S5), while in rootstocks (ZJ_ZJ), *Coniosporium*, *Pleurodesmospora*, *Tilletia*, and *Uwebraunia* increased relative to ZJ, except for *Penicillium*, which decreased (Figure 4E). These fungal shifts suggest that grafting promotes the proliferation of specific genera, potentially enhancing adaptation and physiological responses in tea plants.

### 2.4. Responses of Metabolome, Transcriptome, and Microbiome of Tea Plant to Grafting

**Figure 5:**
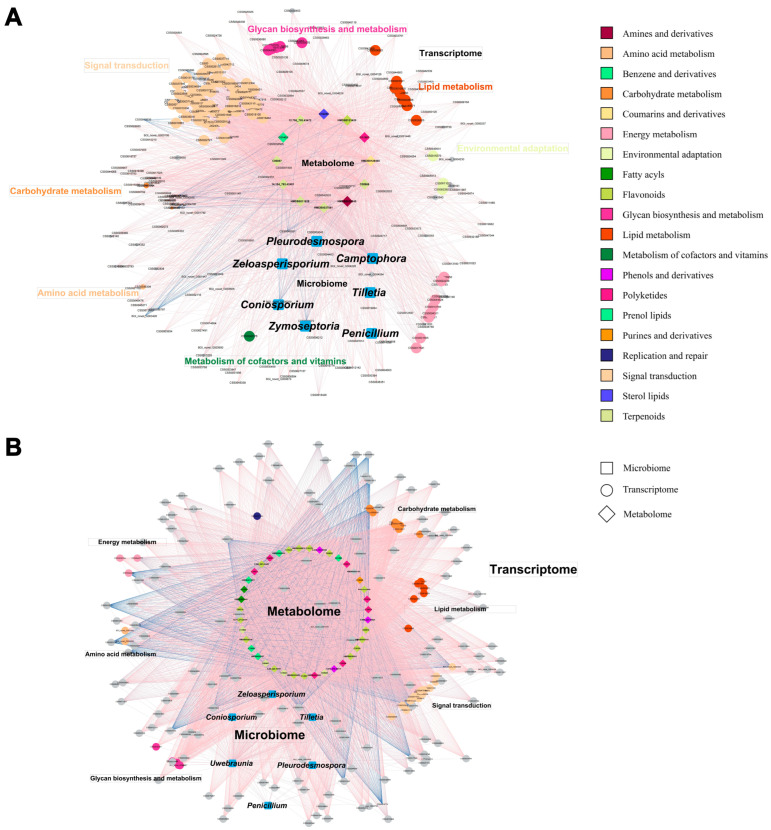
Co-occurrence network analysis of differential species, differential metabolites, and differential genes. (**A**) Difference analysis network of ZJ_YC and YC. (**B**) Difference analysis network of ZJ_ZJ and ZJ. Based on co-occurrence network analysis, key nodes were identified, and a co-occurrence network diagram was constructed based on a Pearson correlation coefficient > 0.07 and a *p*-value < 0.05. Network diagrams illustrating the interactions between the microbiome, transcriptome, and metabolome. Nodes represent different biological entities: squares for the microbiome, circles for the transcriptome, and diamonds for the metabolome. Edges indicate interactions, color-coded by metabolic pathways. Red edges represent positive correlations, while blue edges represent negative correlations.

To elucidate the combined effects of grafting on tea plants, we constructed co-occurrence networks integrating differentially expressed genes (DEGs), microorganisms with altered abundance, and metabolites with varying levels. The networks differed between scions and rootstocks, reflecting distinct regulatory dynamics. In scions, the differential network comprised 12 metabolites, 7 microbial genera, and 251 DEGs (Figure 5A), while in rootstocks, it included 39 metabolites, 6 genera, and 184 DEGs (Figure 5B). A strong association emerged between metabolites and gene expression in both tissues, with signal transduction pathways dominating the networks. In scions, signal transduction genes formed 21.9% of all edges, compared to 7.4% in rootstocks (Table S4), indicating a more pronounced regulatory role in scions. This suggests that grafting activates signal transduction to modulate physiological and metabolic responses, influencing metabolite content in both scion and rootstock.

Focusing on flavonoids, key pathway-related metabolites showed altered levels in both tissues and were linked to signal transduction genes and fungal genera within the networks (Figure 5). These connections highlight how grafting enhances flavonoid metabolism, particularly in rootstocks, through gene–fungi interactions, likely contributing to antioxidant and defense mechanisms. Procrustes analysis confirmed a robust correlation between metabolomics and transcriptomics (Figure S6A), whereas the microbiome–metabolome relationship was weaker for both bacteria and fungi (Figure S6B,C), suggesting that gene expression plays a more direct role in metabolic shifts than microbial changes.

Additional insights emerged from specific metabolite–microbe interactions. Terpenoids, beyond their association with signal transduction genes, positively correlated with fungal genera such as *Zeloasperisporium*, *Coniosporium*, *Tilletia*, and *Penicillium*, implying that fungal abundance shifts may regulate terpenoid levels. Polysaccharide and lipid metabolism pathways were also active in both scions and rootstocks, underscoring the role of energy metabolism and structural adjustments during grafting. In rootstocks (ZJ_ZJ), a core network revealed a dense, complex response involving highly interconnected gene clusters tied to stress response, growth regulation, and metabolic changes (Figure S7). This intricate network suggests that rootstocks undergo substantial physiological reprogramming to support grafting adaptation.

Together, these results demonstrate that grafting coordinates a complex response involving the metabolome, transcriptome, and microbiome, where signal transduction and fungal interactions appear to influence key metabolic changes, notably in flavor-related compounds such as flavonoids and terpenoids.

## 3. Discussion

Tea grafting is an important horticultural practice for the production of tea with high quality, nutrient value, and resilience to multiple environments. In our study, YC served as the scion and ZJ functioned as the rootstock. Through the integrative multi-omics analysis, we revealed how scion and rootstock influenced the resultant tea leaves from the perspectives of metabolic and transcriptomic profiles as well as the microbial community structure.

Secondary metabolites play a pivotal role in tea quality, flavor, and health benefits [4,16]. Grafting can significantly alter the accumulation of secondary metabolites [17]. Cheng et al. investigated the molecular basis of flavonoid accumulation in tea leaves grafted with *Camellia sinensis* var. assamica cv. “Yinghong9” as the rootstock and “Echa 1” as the scion [11]. They found that hetero-grafting (EC1-YH9) increased the total flavonoid and catechin content compared to self-rooted grafting (EC1-EC1) [11]. Tuwei found that grafting scion “YingHong NO. 9” onto various rootstocks (WuLingHong and BaiYeDanCong) led to the differential accumulation of flavan-3-ols and dimeric catechins [9]. Rootstocks like WuLingHong significantly upregulated these metabolites, indicating a rootstock-specific influence on flavonoid biosynthesis pathways. In our study, we found that the grafted tea leaves integrated the metabolic profile of non-grafted plants, mainly driven by the rootstock. We further characterized that the abundance difference in metabolites between non-grafted species was the specific driver for the change in scions and rootstocks. Specifically, we found that the metabolites higher in YC than ZJ would enhance the accumulation of these metabolites in rootstock (ZJ_ZJ). Carbohydrates, phenol lipids, flavonoids, phenols, and derivatives were significantly enhanced in ZJ_ZJ compared to ZJ. Especially, flavonoids were significantly enhanced by grafting. Flavonoids are the vital secondary metabolites of ZJ and play an important role in the flavor quality, tea color, and health benefits of ZJ tea [18,19].

The changes in secondary metabolites are often driven by molecular and physiological interactions between the scion and rootstock [11]. Cheng et al. used metabolomic and transcriptomic analyses to reveal that grafting enhanced 87 metabolites (including flavonoids) and decreased 86 metabolites [11]. This increase was mainly due to the upregulated expression of key genes involved in the flavonoid biosynthesis pathways, including chalcone isomerase, dihydroflavonol 4-reductase, and UDP-glycosyltransferase 75C1 [11]. This indicates that rootstocks influence scion metabolism through transcriptional regulation. In our study, the transcriptomic analysis identified a significant enhancement of *DFR*, the key gene encoding flavanone 4-reductase for synthesizing the apiforol and luteoforol (Figure 3D). At the same time, EC 1.1.1.219 encoding bifunctional dihydroflavonol 4-reductase was significantly enhanced, which enhanced the biosynthesis of leucodelphinidin, leucocyanidin, and leucopelargonidin. We also found that the decreased expression of the catabolism of secondary metabolites plays an important role in the acculturation. Genes including *ANS* and *LAR* encoding anthocyanidin synthase [EC:1.14.20.4] and leucoanthocyanidin reductase [EC:1.17.1.3], respectively, were significantly (*p* < 0.05) suppressed in ZJ_ZJ compared with ZJ. Taken together, tea grafting enhances the accumulation of flavonoids by increasing their biosynthesis and suppressing their biodegradation.

Grafting can alter the microbial composition of plants [20,21]. However, we found that the alpha diversity of the microbial community of grafted tea leaves was only slightly altered, which was driven by the side with the higher alpha diversity. In comparison with YC, the alpha diversity of both bacterial and fungal communities in the grafted tea leaves (ZJ_YC) was slightly increased, mainly due to the influence of ZJ and ZJ_ZJ with higher alpha diversity (Figure 4A and Figure S3A). The slight increase in alpha diversity is probably attributed to the introduction of new microbes from the rootstock with the grafting process [22]. Some previous studies reported that grafting leads to significant shifts in the microbial community structure, as different microbial species establish themselves in the new plant environment [21,22]. In our study, we found that the structure of both bacterial and fungal communities in the drafted tea leaves was not significantly changed (Figure 4B and Figure S3B). This might be attributed to the relative stability of the dominant fungal and bacterial taxa of tea leaves from the scion in our study (Figure 4C and Figure S3C). In summary, the significant change in microbial community structure depends on the colonizing capabilities of newly introduced species and the stability of the native dominant microbes.

Despite the insignificant difference in microbial community structure after grafting, we found that the dominant scion fungal and bacterial taxa still possess different adaptability to the change in microenvironments caused by grafting. Bacteria are not significantly sensitive to the change after grafting. However, drafting significantly enriches the dominant fungal genera including *Camptophora*, *Zeloasperisporium*, and *Zymoseptoria* in scions (ZJ_YC) compared with YC (Figure 4E). The higher abundance of *Camptophora*, *Zeloasperisporium*, and *Zymoseptoria* in ZJ_YC than YC might be due to the decreased content of antibacterial metabolites including flavonoids, phenols, and their derivatives. Flavonoids, phenols, and their derivatives show broad-spectrum antifungal properties via the disruption of cell membranes, the inhibition of fungal enzymes, and the generation of reactive oxygen species [23,24,25,26].

In conclusion, this study reveals that tea grafting drives the shift in secondary metabolites between scions, rootstocks, and non-grafted tea plants. The shifting pattern was driven by the abundance gap of dominant metabolites between the two species. Metabolites lower in the scion-giving plants were enhanced in scions, while metabolites higher in the scion-giving plants were decreased. More importantly, we found that tea grafting considerably enhances the synthesis of flavonoids by upregulating the biosynthetic genes *DFR* and *EC 1.1.1.219* and downregulating the catabolic gene *ANS*. This study provides a novel and deep understanding of the metabolic profile shift by tea grafting and provides a theoretical basis for tea planting and breeding using grafting.

## 4. Materials and Methods

### 4.1. Experimental Design and Sampling

ZJ (*Camellia sinensis* var. assamica cv. Zijuan) is one of the most well-known representative purple tea cultivars with the feature of extraordinary purple shoots [14,18]. It originated in Yunnan Province and has been widely planted in various provinces in China [14,19]. YC (*Camellia sinensis* var. assamica cv. Yuncha1), originated in Yunnan Province, is a tea cultivar with big green leaves with features of strong fertility, good tenderness retention, dense germination, and abundant hairy hairs [15,27]. The tree ages of scions and rootstocks are 7 and 9 years, respectively. In general, the tea plant planted for more than 5–6 years is a mature tea tree. In the maturity of the tea tree, the yield and quality are stable.

In this study, the mature ZJ and YC grafts were used and sampled and sequenced 2 years after grafting. Four experimental groups included the scion group (ZJ_YC), the rootstock group (ZJ_ZJ), and the non-grafted control tea samples ZJ and YC. The experiments were operated in 2021 using cleft grafting. All tea plants were grown in the field of the Tea Research Institute, Yunnan Academy of Agricultural Sciences, Xishuangbanna, China. Each experimental group consisted of 6 biological replicates, and every biological replicate was derived from an individual plant, respectively. Fresh tea leaves with one bud and two leaves were sampled at the same period before and after grafting.

### 4.2. DNA Extraction of Fresh Tea Leaf Microbiome and Amplicon Sequencing

The total DNA (30 ng) was extracted and obtained from fresh tea leaves using the NucleoSpin 96 Soil Kit (MACHEREY-NAGEL, Hilden, Germany), according to the manufacturer’s instructions. The 16S rRNA gene V3–V4 region used the following specific primers: 314F (5′-CCTAYGGGRBGCASCAG-3′) and 806R (5′-GGACTACHVGGGTWTCTAAT-3′), and the fungal internal transcribed spacer 2 (ITS2) region using the forward primer ITS7 (5′-GTGARTCATCRARTYTTTG-3′) and the reverse primer ITS4 (5′-CCTSCGCTTANTDATATGC-3′) were amplified incorporating a 6 bp barcode at the 5′ end for sample identification [28]. PCR amplification was performed in a 25 μL reaction mixture containing 5 μL of HiFi Buffer (5×), 1 μL of dNTPs (2.5 mM), 1 μL of each primer (10 μM), 0.5 μL of KAPA enzyme (5 U/μL), 15.5 μL of ddH_2_O, and 1 μL of template DNA (20 ng/μL). The PCR conditions were as follows: initial denaturation at 98 °C for 5 min; 10 cycles of 98 °C for 30 s, 50 °C for 30 s, and 72 °C for 1 min, followed by 25 cycles of 98 °C for 30 s, 52 °C for 30 s, and 72 °C for 1 min, and a final extension at 72 °C for 5 min. Post-amplification, DNA concentration, and purity were assessed using a Qubit 3 Fluorometer (Thermo Scientific, Wilmington, DE, USA). The quality of extracted DNA was verified via 1% agarose gel electrophoresis. Equimolar concentrations of the purified PCR products were pooled for sequencing using the DNBSEQ-G400 platform [29] at BGI (Shenzhen, China).

### 4.3. RNA Extraction of Fresh Tea Leaves and Library Construction

The RNA of 10 g of fresh tea leaves was extracted using the Biozol Plant RNA Extraction Kit, following the protocol of the manufacturer (Thermo Fisher Scientific, Wilmington, DE, USA). The quantity and quality of RNA were evaluated using a NanoDrop 2000c Spectrophotometer (Thermo Scientific, Wilmington, DE, USA) and agarose gel electrophoresis. Further assessment of RNA samples was conducted with the Agilent Bioanalyzer 2100 system (Agilent Technologies, Santa Clara, CA, USA). mRNA was enriched from the total RNA using oligo (dT) magnetic beads and subsequently fragmented into approximately 200 bp fragments. cDNA was synthesized using a random hexamer primer and purified with magnetic beads. Following end repair and 3′-end adenylation, adaptors were ligated to the cDNA fragments. The resulting fragments were enriched by PCR amplification and purified using magnetic beads. Library quality was evaluated using the Agilent 2100 Bioanalyzer, and the libraries were quantified using the ABI StepOnePlus Real-Time PCR System. The samples were sequenced with the DNBSEQ-G400 platform at BGI-Shenzhen.

### 4.4. Metabolite Extraction and Identification

For metabolite extraction, 5 g of fresh leaf was accurately weighed into a 1.5 mL Eppendorf tube, and 800 μL of pre-cooled extraction solution (methanol: H_2_O = 7:3, *v*/*v*) along with 20 μL of internal standard 1 (IS1) was added. The mixture was vortexed and homogenized using a tissue grinder at 50 Hz for 10 min, followed by ultrasonic treatment in an ice-cold water bath for 30 min. After standing at −20 °C for 1 h, the extraction was centrifuged at 14,000 rpm and 4 °C for 15 min. Subsequently, 600 μL of the supernatant was filtered through a 0.22 μm membrane. A 20 μL aliquot from each sample’s filtrate was pooled to prepare a quality control (QC) sample for assessing the reproducibility and stability of the LC/MS analysis. The filtered samples and the pooled QC sample were transferred to 1.5 mL sample vials for instrument analysis. Metabolite separation and detection were performed using a Waters UPLC I-Class Plus system (Waters, USA) coupled with a Q Exactive high-resolution mass spectrometer (Thermo Fisher Scientific, USA).

The Compound Discoverer 3.3 (https://mycompounddiscoverer.com/, accessed on 13 July 2024) was used for LC-MS data processing to obtain a data matrix table containing information such as metabolite peak area and identification results. An additional database, the BGI Metabolome Database, was used to assist in metabolite identification. Subsequently, the table underwent further information analysis and processing.

### 4.5. Bioinformatics Analysis

For microbiota data, sequences were imported into the QIIME2 (Quantitative Insights into Microbial Ecology Version 2, version 2022.2) pipeline [30]. Primers and barcodes were removed through the Cutadapt plugin. Low-quality sequences were filtered using “qiime quality-filter--p-min-quality 30”. The ASVs mapped to mitochondria and chloroplasts were removed. Based on the DADA2 (Divisive Amplicon Denoising Algorithm 2, version 3.16), amplified subsequence variants (ASVs) and ASV tables were generated with default parameters [31]. Feature sequences with a frequency of ≤4 were discarded. Taxonomic annotation was performed using the UNITE database (https://unite.ut.ee) for fungi, with specific primers for the ITS2 region, and the Silva database (https://www.arb-silva.de) for bacteria, with specific primers for the 16S V3–V4 region.

For transcriptome data, sequence quality control was performed using SOAPnuke (version v1.4.0) [32] with the following steps: (1) removal of reads containing adapter sequences (adapter contamination); (2) removal of reads with more than 5% of unknown bases (N); (3) removal of low-quality reads, defined as reads with more than 20% of bases having a quality score below 15. The parameters used were as follows: -l 15 -q 0.2 -n 0.1. Clean reads were then aligned to the reference genome sequence using Bowtie2 [33], followed by the calculation of gene and transcript expression levels using RSEM (version v2.2.5) [34]. The parameters for Bowtie2 alignment were as follows: -q --phred64 --sensitive --dpad 0 --gbar 99999999 --mp 1, 1 --np 1 --score-min L, 0, -0.1 -p 16 -k 200.

For metabolome data, the mass spectrometry data were imported into Compound Discoverer 3.3 software (Thermo Fisher Scientific, USA). The analysis was conducted using the BMDB (BGI Metabolome Database), mzCloud database, and ChemSpider online database. After processing the mass spectrometry data, a data matrix containing metabolite peak areas and identification results was obtained. This data matrix was then subjected to further information analysis and processing. The results exported from the Compound Discoverer were imported into metaX for data preprocessing and subsequent analysis. The data preprocessing steps include the normalization of the data using the Probabilistic Quotient Normalization (PQN) method to obtain relative peak areas. The correction of batch effects using QC-RLSC (Quality Control-Based Robust LOESS Signal Correction), which applies localized polynomial regression to correct signal drift. The removal of compounds with a coefficient of variation (CV) greater than 30% in the peak areas of all QC samples was achieved.

### 4.6. Statistical Analysis

Alpha diversity was assessed by determining the Shannon index, using the abundance-based coverage estimator (ACE) through the “qiime diversity alpha” command. Principle coordinate analysis (PCoA) was performed based on the Aitchison distance used to characterize beta diversity. The permutational analysis of variance (PERMANOVA) was applied to test group differences based on the distance matrix using “adonis” in a vegan R package (4.1.0) [35]. The relative abundance table of the top 20 species was selected using the maximum abundance ranking method. Differences in alpha diversity were evaluated using the Kruskal–Wallis test (*p*-value < 0.05). The ANCOM2 package was implemented to assess differences in taxa with a detection threshold greater than 0.7 [36]. LEfSe (linear discriminant analysis effect size) was used to test the difference in species abundance with an LDA threshold set to 2 [37]. Biomarkers identified by both LEfSe (LDA > 2.0) and ANCOM2 (detection threshold > 0.7) were selected, and their inter-group abundances were visualized using barplots. Differential genes were identified using DESeq2 (adjusted *p*-value < 0.05), and KEGG enrichment analysis was performed using the phyper function in R software (https://en.wikipedia.org/wiki/Hypergeometric_distribution, accessed on 20 August 2024). Partial least-squares discriminant analysis (PLS-DA) was performed for all metabolites. Annotated differential metabolites were selected based on the criteria of PLS-DA VIP ≥ 1, a fold change (FC) ≥ 2, or a FC ≤ 0.5, and an adjusted *p*-value ≤ 0.05. We then compared these differential metabolites using a Venn diagram and analyzed them with boxplots according to their compound types. Based on their Spearman correlation coefficients (r > 0.7, *p* < 0.05), network analysis was performed among significantly different genes, metabolites, or genera. The network was visualized and analyzed using Cystoscape (v 3.10.1). Other visualizations, except for the network, were performed in the R platform by using the pheatmap or ggplot2 package. A Venn diagram was used to create Venn diagrams. The statistical analysis scripts are available on GitHub: https://github.com/GoGoGao/tea_grafting.

## Data Availability

The raw RNA-seq and amplicon sequencing data presented in this study can be found in NCBI Sequence Read Archive (SRA), and the accession number is PRJNA1146230. The metabolomics data have been uploaded to the NGDC database, and the project number is: PRJCA029044.

## Supplementary Materials

The following supporting information can be downloaded at: https://www.mdpi.com/article/10.3390/plants14081176/s1, Figure S1: Yuncha1 was grafted on the Zijuan rootstock as scion; Figure S2: The number of metabolites categorized into different metabolic pathways; Figure S3: (A) The volcano plot of transcriptional differences between YC and ZJ_YC, identifying 8558 genes that are upregulated and 8493 that are downregulated. (B) The volcano plot of transcriptional differences between ZJ and ZJ_ZJ, with 6918 genes upregulated and 6795 genes downregulated. (C) The heatmap displays transcriptional different genes involved in the pathways responsible for the synthesis of differential metabolites, highlighting distinct expression patterns across the four groups: YC, ZJ_YC, ZJ, and ZJ_ZJ. Pathways related to metabolism (KEGG level 1), including amino acid metabolism, carbohydrate metabolism, energy metabolism, and lipid metabolism, contain the most differentially expressed genes; Figure S4: (A)The boxplot comparison of bacterial alpha diversity across the groups YC, ZJ_YC, ZJ, and ZJ_ZJ. (B) Principal Coordinates Analysis (PCoA) plot based on Aitchison distance on bacterial ASV level. The square represents YC and the triangle represents ZJ. (C) Hierarchical clustering based on Bray–Curtis distance and relative abundance barplot of the top 20 bacterial genera. (D) Venn diagrams displaying the number of bacterial genera between YC and ZJ_YC and between ZJ and ZJ_ZJ; Figure S5: ANOSIM analysis performed to assess the significance of the community structure differences in fungi and bacteria between YC and ZJ after grafting at the genus level; Figure S6. Procrustes analysis for the relationship between metabolomics and transcriptomics (A), metabolomics and bacteria (B), and metabolomics and fungi (C). A *p* value < 0.05 was considered significant; Figure S7: ZJ_ZJ core co-occurrence network analysis of differential metabolites and differential genes. Network diagrams illustrate interactions between the transcriptome and metabolome. Nodes represent different biological entities: circles for the transcriptome and diamonds for the metabolome. Edges indicate interactions, color-coded by metabolic pathways. Red edges represent positive correlations, while blue edges represent negative correlations; Table S1: Functional classification and number of known metabolites detected in the metabolomics analysis; Table S2: The table of differential metabolites screened using VIP > 1, FC > 2 | FC < 0.5, and q < 0.05; Table S3: Differential genes in differential metabolic pathways between ZJ_ZJ and ZJ; Table S4: Node type number belonging to different gene function or metabolic class.
