# Supplementary material for: Multi-Omics Analysis Revealed the Accumulation of Flavonoids and Shift of Fungal Community Structure Caused by Tea Grafting (Camellia sinensis L.)
Source: Plants (Basel). 2025 Apr 10;14(8):1176. doi: 10.3390/plants14081176 (PMC12030563; doi:10.3390/plants14081176)
Supplement: Supplementary file 1 [file plants-14-01176-s001.zip › Supplementary File/Supplementary Figures.docx]

**Supplementary material**


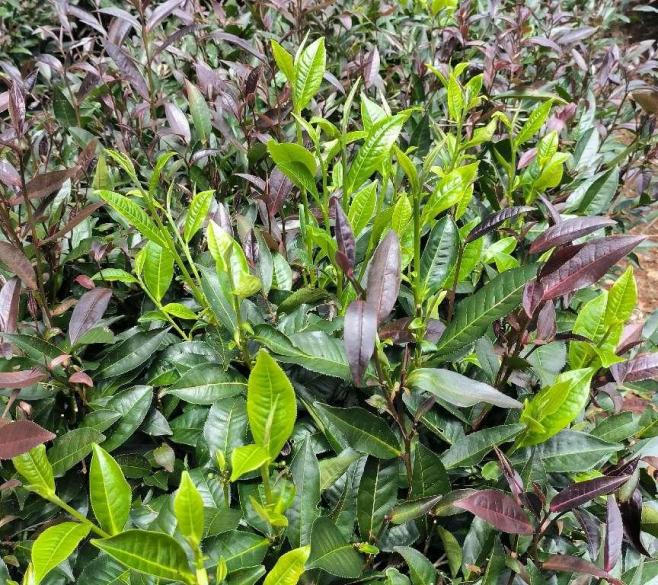


**Figure S1.** Yuncha1 was grafted on the Zijuan rootstock as scion.

**Figure S2**. The number of metabolites categorized into different metabolic pathways.


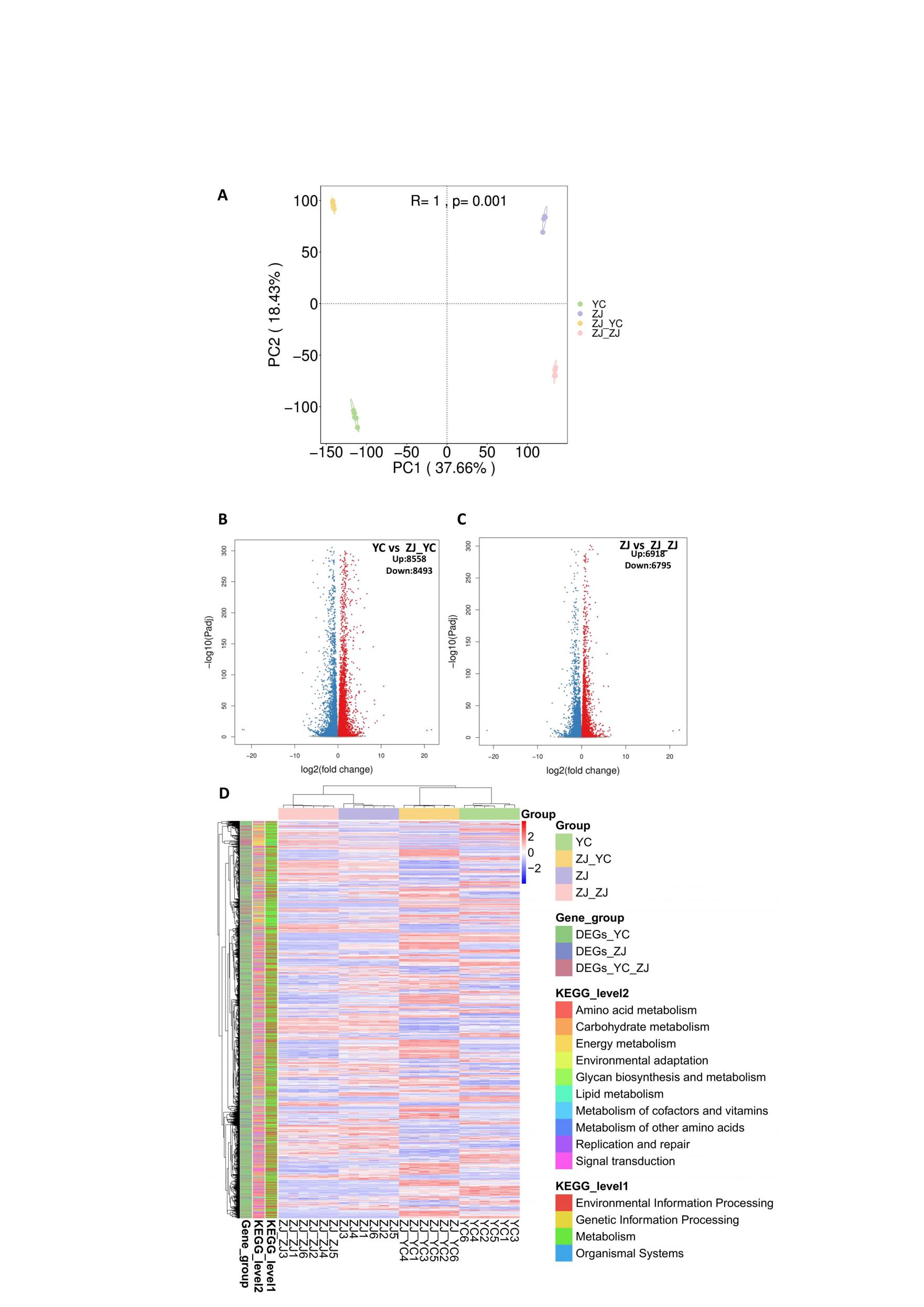


**Figure S3**. (A) The volcano plot of transcriptional differences between YC and ZJ_YC, identifying 8,558 genes that are up-regulated and 8,493 that are down-regulated. (B) The volcano plot of transcriptional differences between ZJ and ZJ_ZJ, with 6,918 genes upregulated and 6,795 genes down-regulated. (C) The heatmap displays transcriptional different genes involved in the pathways responsible for the synthesis of differential metabolites, highlighting distinct expression patterns across the four groups: YC, ZJ_YC, ZJ,ZJ_ZJ. Pathways related to metabolism (KEGG level 1), including amino acid metabolism, carbohydrate metabolism, energy metabolism, and lipid metabolism, contain the most differentially expressed genes.

**Figure S4**. (A)The boxplot comparison of bacterial alpha diversity across the groups YC, ZJ_YC, ZJ, and ZJ_ZJ. (B) Principal Coordinates Analysis (PCoA) plot based on aitchison distance on bacterial ASV level. Square represents YC and triangle represents ZJ. (C) Hierarchical clustering based on Bray-Curtis distance and relative abundance barplot of the top 20 bacterial genera. (D) Venn diagrams displaying the number of bacterial genera between YC and ZJ_YC, and between ZJ and ZJ_ZJ.

**Figure S5**.ANOSIM analysis performed to assess the significance of the community structure differences in fungi and bacteria between YC and ZJ after grafting at genera level.


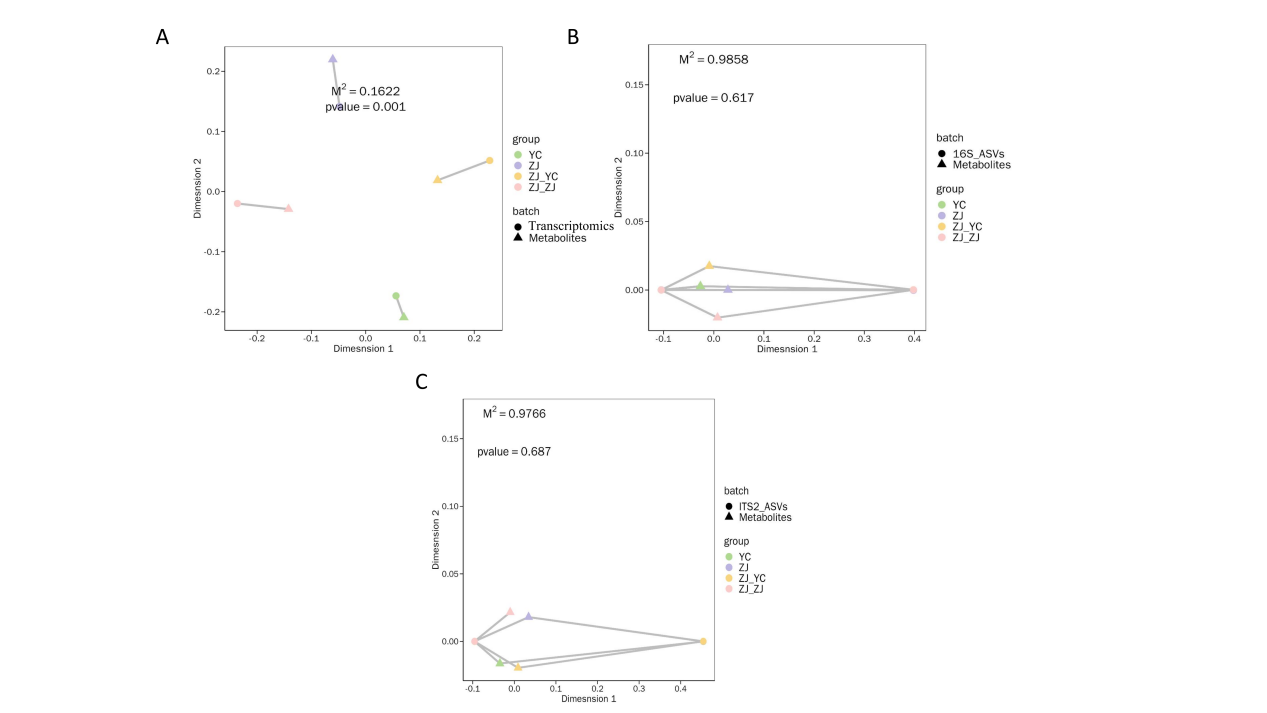


**Figure S6**. Procrustes analysis for relationship between metabolomics and transcriptomics (A), metabolomics and bacteria (B), metabolomics and fungi (C). P value＜0.05 was considered significant.

**Figure S7**. ZJ_ZJ core co-occurrence network analysis of differential metabolites and differential genes. Network diagrams illustrate interactions between the transcriptome and metabolome. Nodes represent different biological entities: circles for the transcriptome and diamonds for the metabolome. Edges indicate interactions, color-coded by metabolic pathways. Red edges represent positive correlations, while blue edges represent negative correlations.
